# Pilot Newborn Screening for Vitamin B12 Deficiency in the Czech Republic: Results and Detailed Studies on Identified Babies and Their Mothers

**DOI:** 10.3390/ijns12020030

**Published:** 2026-05-05

**Authors:** Samuel Stanovský, Josef Bártl, Petr Chrastina, Viktor Kožich, Jakub Krijt, Kristýna Nelicová, Jitka Sokolová, Truong An Nguyen, Richard Plavka, Květa Pelinková, Drahomíra Springer, Klára Berková, Zbyněk Straňák, Jan Janota, Katarína Tichá, Jiří Zach, Tomáš Honzík

**Affiliations:** 1 Department of Pediatrics and Inherited Metabolic Disorders, First Faculty of Medicine, Charles University and General University Hospital, 128 08 Prague, Czech Republic; samuel.stanovsky@vfn.cz (S.S.); josef.bartl@vfn.cz (J.B.); petr.chrastina@vfn.cz (P.C.); viktor.kozich@vfn.cz (V.K.); jakub.krijt@vfn.cz (J.K.); kristyna.nelicova@vfn.cz (K.N.); jitka.sokolova@vfn.cz (J.S.); 2 Department of Gynecology, Obstetrics and Neonatology, First Faculty of Medicine, Charles University and General University Hospital, 128 08 Prague, Czech Republic; truongan.nguyen@vfn.cz (T.A.N.); richard.plavka@vfn.cz (R.P.); 3 Institute of Medical Biochemistry and Laboratory Diagnostics, First Faculty of Medicine, Charles University and General University Hospital, 128 08 Prague, Czech Republic; kveta.pelinkova@vfn.cz (K.P.); drahomira.springer@vfn.cz (D.S.); 4 Department of Neonatology, Third Faculty of Medicine, Charles University and Institute for the Care of Mother and Child, 147 00 Prague, Czech Republic; klara.berkova@upmd.eu (K.B.); zbynek.stranak@upmd.eu (Z.S.); 5 Neonatal Unit, Motol and Homolka University Hospital, 150 06 Prague, Czech Republic; jan.janota@fnmotol.cz (J.J.); katarina.ticha@fnmotol.cz (K.T.); 6 Neonatal Unit, Thomayer University Hospital, 140 59 Prague, Czech Republic; jiri.zach@ftn.cz

**Keywords:** vitamin B12, holoTC, active B12, methylmalonic acid, homocysteine, B12 deficiency, CobaSorb, newborn screening, chronic gastritis

## Abstract

Neonatal vitamin B12 (B12) deficiency can cause neurodevelopmental harm, and newborn screening (NBS) may enable early detection and treatment. We conducted a multicenter pilot project in four Prague university hospitals between 1 June 2022 and 30 June 2025. Algorithms included the determination of propionylcarnitine-derived primary markers using flow-injection tandem mass spectrometry and second-tier methylmalonic acid (MMA), with total homocysteine measured only when MMA was increased. Of 34,302 screened newborns with consent, 1365 (3.98%) triggered second-tier testing; 9 had MMA > 2.5 µmol/L, of which 8 met the case definition after confirmatory testing, giving a birth frequency of 1:4228 (95% CI 1:2176–1:9931). Positive predictive value was 0.59% (95% CI 0.25–1.15%) and 88.89% (95% CI 51.75–99.72%) for the primary test and second-tier MMA, respectively, with a false positive rate of 0.00292% (95% CI 0.000074–0.01625%). All affected infants were treated orally with cyanocobalamin. Maternal work-up identified confirmed B12 deficiency in four of eight mothers and premalignant gastric changes in two of four positive women. These data support the feasibility, low cost, and clinical utility of incorporating B12 deficiency into Czech NBS, with benefits extending beyond newborn health.

## 1. Introduction

**Figure 1:**
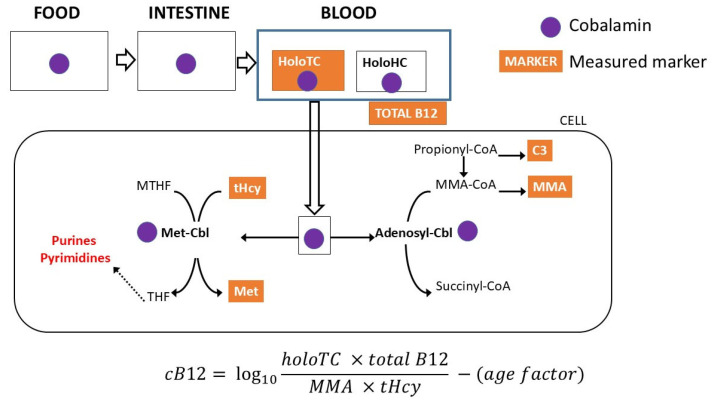
Cobalamin metabolism and markers of B12 deficiency. Cbl—cobalamin; cB12—combined B12 factor (formula according to Fedosov et al. [7]); HoloHC—holohaptocorrin; HoloTC—holotranscobalamin; Met—methionine; MMA—methylmalonic acid; MTHF—methyltetrahydrofolate; tHcy—total homocysteine; THF—tetrahydrofolate.

Vitamin B12 (B12) or cobalamin (Cbl) is a water-soluble vitamin naturally present in animal-source foods (meat, fish, eggs, dairy) [1]. Exclusively breastfed infants depend on prenatally accumulated hepatic Cbl stores and postnatal supply of B12 from breast milk [2,3,4]. Intracellularly, Cbl serves as a cofactor for methylmalonyl-CoA mutase and methionine synthase in the form of adenosyl-Cbl and methyl-Cbl, respectively (see Figure 1) [5]. The estimated adequate intake (AI) for B12 is approximately 0.4 µg/day in neonates and infants, whereas the recommended dietary allowance is 2.4 µg/day in adolescents and adults and 2.6–2.8 µg/day in pregnant and lactating women [6].

Adequate B12 status is assessed by four main biomarkers. In circulation, B12 is present as holotranscobalamin (holoTC) and holohaptocorrin. Only holoTC (active B12) represents the biologically available fraction [8,9]. Routine biochemical assessment uses total B12 and/or holoTC, while the functional status of Cbl-containing enzymes methylmalonyl-CoA mutase and methionine synthase is reflected by serum methylmalonic acid (MMA) and total homocysteine (tHcy), respectively [10,11]. There is no universally accepted biochemical definition of B12 deficiency. In exclusively breastfed infants, holoTC physiologically falls at 4–6 months [12]. MMA is a sensitive marker of intracellular vitamin B12 deficiency. In the first year of life, the upper reference limit is typically 700–1000 nmol/L, with a transient rise to as much as 2000 nmol/L between 4 and 6 months of age. After 12 months, MMA generally decreases to ≤270–300 nmol/L [13,14,15]. In contrast, tHcy is less sensitive and less specific than MMA, but it is widely available [16,17]; typical upper reference limits are about 10.0 µmol/L in children and 15.0 µmol/L in adults [18,19,20]. Serum markers (total B12 and holoTC) and functional markers of B12 deficiency (MMA and tHcy) can be used for calculating combined B12 factor (cB12) [7]; cB12 allows for assessing the degree of B12 deficiency.

B12 deficiency is a frequent and often unrecognized clinical condition. In neonates and infants, the predominant cause is an in-utero acquired deficiency due to unrecognized maternal deficiency resulting from malabsorption (e.g., chronic or autoimmune/metaplastic atrophic gastritis) or low dietary intake (e.g., vegan diet without adequate supplementation). Other etiologies include delayed introduction of complementary food, and rarely, inherited disorders of B12 transport or intracellular processing [21]. In older children and adults, insufficient dietary intake, gastrointestinal disease (coeliac disease, Crohn’s disease, post-resection of the terminal ileum, pancreatic insufficiency, hypo-/achlorhydria, or proton pump inhibitor use), and inherited transport defects are among typical causes [22]. Cbl deficiency impairs DNA synthesis and myelination, leading to macrocytic/megaloblastic anemia and various neuropsychiatric symptoms, respectively.

B12 deficiency in infants may manifest with failure to thrive and poor feeding, multiple neurological abnormalities (lethargy, irritability, hypotonia, absence of social smile, developmental delay or regression, seizures, demyelination); anemia and glossitis may occur [23,24,25,26,27,28]. Macrocytic anemia is not an obligatory finding, presenting in only 28% of infants in our previously reported cohort [21]. It was shown that early therapy is associated with better neurodevelopmental outcomes, supporting NBS for this condition [29,30,31]. Management of B12 deficiency in infants is straightforward and effective, typically based on administration of hydroxo-Cbl or cyano-Cbl orally, although pediatric-specific national or international guidelines are not well developed in contrast to the existing adult guidelines [32]. Intramuscular therapy in infants is rarely needed because disorders causing malabsorption are infrequent in this age group [33,34].

In some programs, B12 deficiency might be detected via NBS for other genetic conditions manifesting with elevated propionylcarnitine (C3) [35,36,37,38,39]. C3-derived markers and methionine (Met) are biochemically suitable first-tier newborn screening markers for vitamin B12 deficiency because impaired vitamin B12–dependent metabolism leads to C3 accumulation and disturbed Metsynthesis. Secondary markers such as MMA and tHcy are particularly useful because they more directly reflect intracellular functional vitamin B12 deficiency and can therefore improve the specificity of the screening algorithm. Although most NBS programs do not include B12 deficiency as a primary or secondary target, a recent study by Mütze et al. [31] demonstrates the benefit of screening for B12 deficiency. Inspired by the Catalonian NBS program [36], our pilot study estimated the birth frequency of neonatal B12 deficiency and evaluated the feasibility of nationwide Czech NBS for this condition.

## 2. Materials and Methods

### 2.1. Study Site and Ethics

Between 1 June 2022 and 30 June 2025, a pilot screening program for B12 deficiency was conducted in four Prague university hospitals: the General University Hospital, Thomayer University Hospital, Motol University Hospital, and the Institute for the Care of Mother and Child. The study was approved by the Ethics Committee of the General University Hospital (approval number EK VFN 31/21). All samples were analyzed at the Diagnostic Laboratories for Inherited Metabolic Disorders, Department of Pediatrics and Inherited Metabolic Disorders, First Faculty of Medicine, Charles University, and General University Hospital. At study collaborating sites, consenting mothers received an informed consent form to enroll their newborns in the pilot screening program for B12 deficiency. Enrolment required no additional blood draw; testing was performed in DBS used for the routine NBS program for 20 conditions. Blood was collected between 48 and 72 h of life.

### 2.2. DBS Primary Markers Analysis

A single 3.2 mm (1/8 inch) disc was punched from each DBS card. Primary screening markers were analyzed using a routine newborn screening method based on flow-injection tandem mass spectrometry (FIA–MS/MS). For this purpose, we used a commercial derivatized kit (MassChrom^®^ Amino Acids and Acylcarnitines from Dried Blood with derivatization-buthylation of the analytes; Chromsystems, Gräfelfing, Germany).

All measurements were performed on triple–quadrupole tandem mass spectrometers manufactured by SCIEX. In our FIA–MS/MS method, a parallel scanning approach was employed, using precursor-ion scanning for acylcarnitines (+Prec 85), neutral-loss scanning (+NL 102), and multiple-reaction monitoring (MRM) transitions for amino acids. Quantification was performed using the appropriate internal standards in accordance with the manufacturer’s instructions. The total analysis time per sample was 35 s.

For the identification of B12 deficiency, we used the following primary markers: C3, the propionylcarnitine/acetylcarnitine ratio (C3/C2), Met, and the propionylcarnitine/methionine ratio (C3/Met); for decision limits, see Section 3. A single abnormal marker was sufficient to classify a result as a positive screening result.

### 2.3. DBS Secondary Markers Analysis

Second-tier tests were carried out on a triple–quadrupole tandem mass spectrometer following chromatographic separation on an analytical column; tests were developed as in-house methods.

An unpublished in-house developed method for MMA analysis was used. A single 3.2 mm (1/8 inch) disc sample was extracted into an acetonitrile/water mixture (70:30, *v*/*v*) on a laminar shaker for 30 min. The extraction solvent contained the internal standard d_3_-MMA (1 µmol/L) and 0.1% formic acid. The extract was subsequently filtered using a 96-well filter plate and analyzed by liquid chromatography–tandem mass spectrometry (LC–MS/MS). The analysis was performed on a SunFire C8 chromatographic column (3.5 µm, 4.6 × 100 mm; Waters, Milford, MA, USA) at a flow rate of 0.55 mL/min under isocratic conditions. The mobile phase consisted of 80% acetonitrile and 20% water containing 0.1% formic acid, and the injection volume was 5 µL. MMA was separated from its isomer succinic acid with retention times of 1.9 and 3.0 min for succinic acid and MMA, respectively. During the 6 min run, MRM transitions were monitored in negative ionization mode for MMA (*m*/*z* 117 → 73) and d_3_-MMA (*m*/*z* 120 → 76). Basic analytical performance characteristics were assessed as part of method validation. The intra-assay coefficient of variation (CV) was below 6%, the inter-assay CV was below 10%, and recovery ranged from 95% to 103%. The limits of detection and quantification were 0.5 and 1.7 µmol/L, respectively.

For the determination of tHcy, we used a modification of a previously published method [40]. Another single 3.2 mm (1/8 inch) disc was extracted for 50 min on an orbital shaker at ambient temperature using the same solvent as above, which was supplemented with the reducing agent dithiothreitol (50 mmol/L) and the internal standard d_8_-homocystine (0.25 µmol/L). Separation was carried out under the same chromatographic conditions, with detection in positive ionization mode using the following MRM transitions: *m*/*z* 136.0 → 90.1 for tHcy and *m*/*z* 140.1 → 94.1 for d_4_-Hcy. The limit of quantification for tHcy was 1.1 µmol/L. The method was internally validated and showed satisfactory precision and accuracy: intra-assay and inter-assay CV were below 10%, and recovery ranged from 80% to 90%.

Quantification of MMA and tHcy was achieved using analyte-specific calibration curves prepared in the DBS matrix. Calibration points for MMA and tHcy were prepared by spiking the analytes into anticoagulated whole blood prior to spotting onto DBS cards. For MMA, spikes ranged from 0.2 to 16 µmol/L. DBS matrix for tHcy determination contained spike concentrations from 5 to 100 µmol/L of the disulfide homocystine; both the spiked homocystine and internal standard d_8_-homocystine were subsequently reduced during the analytical procedure. To further verify the measurements in the DBS matrix, commercially available DBS control materials were included in each analytical series at two concentration levels. Recovery from the DBS matrix was assessed during method validation and was found to be within the acceptable range, as mentioned above. MMA was used as the principal second-tier marker; tHcy was measured only in the case of an increased MMA concentration. Recall decisions were based exclusively on MMA concentrations. 

### 2.4. Clinical and Laboratory Investigation of Positively Screened Newborns and Their Mothers

After a positive screening result, newborns and their mothers were invited for outpatient evaluation. Both the newborn and the mother were clinically examined. In mothers, dietary habits from the beginning of pregnancy were assessed using a Food Frequency Questionnaire (FFQ) (Supplementary Material S1 FFQ), and vitamin supplement use was recorded. Biochemical B12 deficiency was defined as (1) at least one serum marker (total B12 or holoTC) was decreased and simultaneously, and (2) at least one intracellular functional marker (MMA or tHcy) was elevated.

In newborns, confirmatory laboratory investigations included a complete blood count with differential, serum total B12 (electrochemiluminescence immunoassay; Elecsys Vitamin B12 II, COBAS 8000, module e811, Roche Diagnostics, Basel, Switzerland), serum holoTC (chemiluminescence immunoassay; Active-B12, Alinity I, Abbott, Abbott Park, IL, USA), and serum folate (electrochemiluminescence immunoassay; Elecsys Folate III, COBAS 8000, module e811, Roche Diagnostics). Serum/plasma amino acid profiles were analyzed using ion-exchange chromatography on an automatic amino acid analyzer with post-column ninhydrin derivatization (AAA 400 automatic amino acid analyzer, INGOS, Praha 4-Komořany, Czech Republic). Profiles of urinary organic acids were determined using gas chromatography–mass spectrometry (Thermo Fisher, Waltham, MA, USA) after conversion to the corresponding silyl derivatives. Serum/plasma MMA concentrations were measured by LC–MS/MS (ClinMass LC-MS/MS Complete kit for Metylmalonic acid in Serum/Plasma/Urine, RECIPE, Munich, Germany); according to manufacturer’s manual, the intra-assay CV is below 6%, the inter-assay CV is below 6.1%, and recovery ranges from 91% to 116%; limits of detection and quantification are 15 and 25 µmol/L, respectively. tHcy was determined using a commercial enzymatic photometric assay (HOMOCYSTEIN 2R, LS 2 Liquid Stable Reagent Kit, Axis-Shield, Dundee, UK) on an ERBA XL 200 analyzer; according to manufacturer’s manual, the intra-assay coefficient of variation (CV) is below 4%, the inter-assay CV is below 6.6%, and the limit of detection is 0.6 µmol/L. The combined factor cB12 was calculated using the formula of Fedosov et al. [7].

In mothers, we additionally measured erythrocytic folate by electrochemiluminescence immunoassay (Folate RBC Hemolyzing Reagent plus Elecsys Folate III, COBAS 8000, module e811, Roche Diagnostics) and a panel of laboratory markers of atrophic gastritis as follows. Gastric parietal cell antibodies (GPCAb) were assessed by indirect immunofluorescence (Mosaic Basic Profile FA 1800-2010-2, SPRINTER XL, Euroimmun Medizinische Labordiagnostika, Lübeck, Germany). Anti-intrinsic factor antibodies (anti-IF) were measured by fluoroenzyme immunoassay (EliA Intrinsic Factor Well 14-5668-1, Phadia 250, Thermo Fischer Scientific), as were anti-H^+^/K^+^ ATPase antibodies (EliA Parietal Well 14-5669-1, Phadia 250, Thermo Fischer Scientific). Serum gastrin was analyzed by chemiluminescence (Gastrin-17, Maglumi 800, Snibe Diagnostic, Shenzhen, China). Pepsinogen I and pepsinogen II were measured using an enzyme-linked immunosorbent assay (ELISA; Pepsinogen I [601010.01] and Pepsinogen II [601020.02], Biohit, Helsinki, Finland) on an SLT Spectra microplate reader (SLT Lab Instruments, Ulsan, Republic of Korea), and the pepsinogen I/II ratio was calculated.

### 2.5. Maternal Dietary Patterns During Pregnancy

We retrospectively assessed maternal dietary habits using a Food Frequency Questionnaire (FFQ) modified from publications of Wozniak and Eilander [41,42]. We asked: “Which of the following options best describes your dietary habits from the time you found out you were pregnant? Please provide an average estimate for the entire period during which you have known you were pregnant.” Based on FFQ responses, we classified each woman into one of five diet categories: omnivore, flexitarian, pescatarian, vegetarian, or vegan (see Supplementary Material S1 FFQ).

### 2.6. B12 Absorption Assessment—The CobaSorb Test

The test was adapted from an earlier report by Hvas et al. [43] by using magistrally prepared CobaSorb syrup; see Supplementary Material S2. In mothers, we followed the previously published regimen—9 µg of cyano-Cbl three times daily for 2 days [43]. In newborns, we administered ~10 times the adequate intake of B12: 1.8 µg of B12 syrup three times daily for 2 days (see Supplementary Material S2, Magistral Formula of B12 Syrup), analogous to the ~10 times RDA used in adults. The test was interpreted as a possible malabsorption when the difference between post- and pre-dose holoTC concentrations was <10 pmol/L, or when the post/pre-dose ratio was <1.22.

### 2.7. Use of AI

For manuscript preparation and language editing, we used the large language model ChatGPT, version GPT-5.5 Thinking (OpenAI, San Francisco, CA, USA).

## 3. Results

### 3.1. Study Cohort and Timeliness of Investigations

Over the pilot project period, we received 40,245 DBS samples; consent for participation in the pilot program was available for 34,302 (85.23%) samples. The median age at DBS collection was 3 days; transit time ranged from 1 to 3 days. The median age at primary marker testing was 5 days, and second-tier marker testing was performed within 1 to 7 days after detecting an abnormal first-tier result. The median age of newborns at the first clinical assessment was 18 days, while the median age of their mothers was 35.1 years.

### 3.2. Cut-Offs

For identifying B12 deficiency, we used modified Catalonian algorithms [36]. Since the routine Czech NBS program does not screen for C3-detectable disorders, we first analyzed the distribution of related markers in de-identified acquired MS/MS data from 15,000 DBS. In the next step, we set the cut-offs with corresponding percentiles as follows: C3 > 3.8 μmol/L (96th percentile), C3/C2 > 0.3 (99.8th percentile), C3/Met > 0.5 (99.8th percentile), and Met < 7 μmol/L (1st percentile). The cut-offs were set to balance sufficient sensitivity and an acceptable workload. Our cut-offs were approximately comparable to those used in the Catalan algorithm.

**Figure 2:**
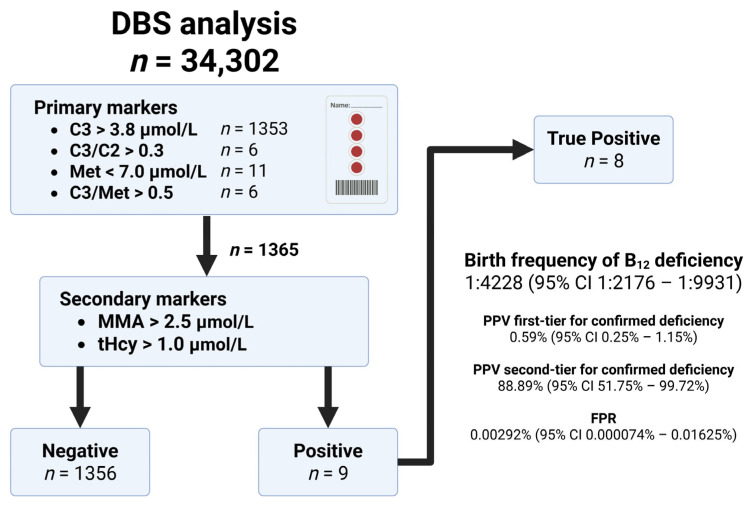
Algorithm for the detection of B12 deficiency and results of newborn screening. C3—propionylcarnitine; C3/C2—propionylcarnitine/acetylcarnitine ratio; C3/Met—propionylcarnitine/methionine ratio; DBS—dry blood spot; FPR—false positive rate; Met—methionine; MMA—methylmalonic acid; PPV—positive predictive value; tHcy—total homocysteine.

A single positive primary marker was sufficient to initiate second-tier testing. As the second-tier marker, we used MMA > 2.5 μmol/L; in such cases, tHcy was also determined (Figure 2).

The complete biochemical results of the first-tier and second-tier tests of recalled newborns are presented in Table 1.

### 3.3. Screening Results

Among screened samples in our cohort, 1365 (3.98%) DBS had an abnormal primary marker and were subject to second-tier MMA testing. The most frequently detected abnormal primary marker was C3 (Figure 2). Nine infants had MMA > 2.5 µmol/L, of whom eight met the case definition (see Table 2), yielding an incidence of 1:4228 (95% confidence interval—CI 1:2176–1:9931). The positive predictive value (PPV) of the first-tier screen for confirmed neonatal vitamin B12 deficiency was 0.59% (95% CI 0.25–1.15%). For a positive second-tier MMA result, the PPV was 88.89% (95% CI 51.75–99.72%). The false positive rate (FPR) was 0.00292% (95% CI 0.000074–0.01625%).

### 3.4. Comparison of C3-Derived and Met-Derived Markers Strategy

**Figure 3:**
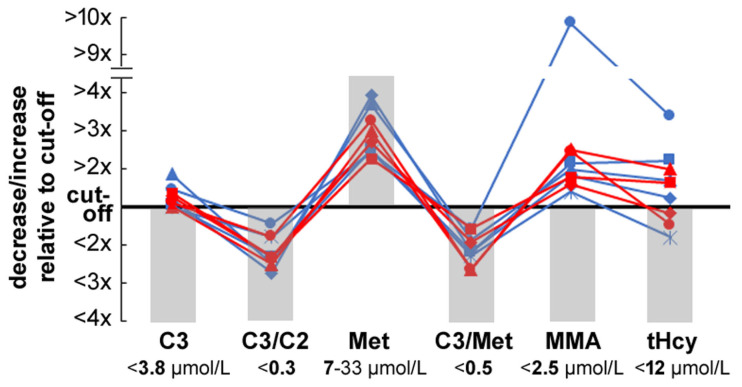
Distribution of primary and second-tier screening marker values in nine newborns recalled after a positive screening result. C3—propionylcarnitine; C3/C2—propionylcarnitine/acetylcarnitine ratio; C3/Met—propionylcarnitine/methionine ratio; MMA—methylmalonic acid; tHcy—total homocysteine; grey bars—physiological reference range; *y*-axis—relative value to cut-off; red—girls; blue—boys.

We also compared the detection performance of C3-derived markers with Met-derived markers for remethylation disorders (Figure 3). None of the infants identified by the C3-derived algorithm would have been detected by the Met-based markers used to screen for remethylation disorders.

### 3.5. Confirmatory Testing in Newborns and Mothers

The results of all confirmatory tests are displayed in Table 2. Of nine recalled newborn cases, seven had decreased serum Cbl markers and elevated MMA, confirming B12 deficiency. Newborn number 5 had supraphysiological concentrations of serum B12 markers, indicating possible B12 administration prior to confirmatory testing. Nevertheless, the subject was classified as functionally deficient due to the high elevation of MMA; other causes of MMA elevation (e.g., CKD) were excluded. One case was false positive, with C3 just close to the cut-off value. Although the cB12 index has not been validated for newborns, all positive cases (except number 5) had this parameter substantially decreased below the threshold for B12 deficiency (<−1.5) [7].

### 3.6. B12 Absorption Testing

We offered each newborn–mother pair the CobaSorb B12 absorption test as part of the evaluation. All newborns absorbed adequately. Of the seven mothers who underwent CobaSorb, malabsorption was proven in two of them, while one woman had borderline absorption.

### 3.7. Assessment of Maternal B12 Status

Four of eight mothers of newborns with confirmed B12 deficiency were also B12 deficient, while B12 deficiency was not confirmed in the remaining women. Maternal characteristics, CobaSorb results, dietary patterns, and pregnancy supplement use with per-kilogram B12 and folate doses are summarized in Table 3. We referred women for upper gastrointestinal endoscopy with biopsy if either (1) the CobaSorb test demonstrated malabsorption, or (2) laboratory criteria for suspected chronic gastritis were met—defined as elevated gastrin or low pepsinogen I together with positivity for at least one antibody: GPCAb, anti-IF, or H^+^/K^+^ ATPase. Systematic maternal work-up following NBS also revealed premalignant gastric mucosal changes in two women, both of whom were enrolled in regular endoscopic surveillance; in one of them, an early-stage malignancy was subsequently detected.

### 3.8. Newborn Deficiency Treatment

After a deficiency was confirmed, we initiated treatment in all affected newborns. Hydroxocobalamin is not routinely available in the Czech Republic, requires approval by a medical reviewer, and is reimbursed only under a special, insurer-approved payment arrangement. Therefore, each infant received an orally administered, magistrally prepared B12 cyano-Cbl syrup at a dose of 12 µg per day for 6 weeks. After 6 weeks, the dose was reduced to 12 µg every other day. Therapy continued until complementary feeding was introduced in the fifth month of life.

### 3.9. Additional Cost of the Laboratory B12 Deficiency Screening

Determination of C3-derived markers incurred no additional cost, as the analysis was performed on the routinely collected DBS, and C3-related markers were filtered out before the pilot project began. The unit price of a single second-tier assay is 987 CZK (approximately 40.59 EUR as of 21 April 2026), and it was performed for 1365 samples. Spread across 34,384 newborns, this corresponds to an incremental cost of 39.2 CZK (approximately 1.61 EUR as of 21 April 2026) per newborn.

## 4. Discussion

Our study demonstrated a relatively high birth frequency of 1:4228 (95% CI 1:2176–1:9931) of B12 deficiency in newborns delivered in several Prague hospitals, which is comparable to the incidence of HPA/PKU in the Czech Republic (1:4960). Neonatal B12 deficiency in Czechia has a frequency similar to other European countries, i.e., 1:9600 in Germany [31]; approximately 1:5000 in Italy [38]; 1:3000 in Estonia [37]; and approximately 1:2000 in Spain [36]. We hypothesize that the use of different cut-off values and differences in dietary patterns contribute to the variability among countries.

NBS for Cbl deficiency may be implemented into existing programs using various strategies with different diagnostic yields: a Met-based approach (e.g., Met and Met/Phe) aimed primarily at isolated remethylation defects, or a C3-based approach for organic acidurias and combined Cbl disorders with second-tier confirmatory testing (typically MMA, often with methylcitrate) [31,44,45]. The Met-based approach appears to be less sensitive for both the genetically determined combined Cbl disorders [46] as well as for the acquired B12 deficiency (birth frequency of B12 deficiency in the routine Czech Met-based strategy for remethylation defects was 1:223,000; unpublished data). In our pilot study, we used more sensitive C3-derived markers and cut-offs similar to the published Cbl-deficiency workflows [36,47]. C3 is a sensitive marker that may also be increased in propionic acidemia, methylmalonic acidemia, and intracellular Cbl processing disorders. According to CLIR data, C3 alone provides higher sensitivity for detecting B12 deficiency than the C3/C2 ratio [48]. Incorporating second-tier MMA analysis from the same DBS substantially improves PPV and enables early detection of both nutritional B12 deficiency and clinically significant inborn errors of metabolism [31].

It is rarely reported that the NBS for B12 deficiency may have a secondary public health benefit, namely, the detection of maternal B12 deficiency [38,39]. Our findings in mothers numbered 5–7 and 9 suggest that apparently adequate maternal Cbl status in pregnancy may not always ensure sufficient B12 supply to the infant [49,50]. While folate supplementation is routinely emphasized in pregnancy, counselling and routine assessment of maternal Cbl intake/status are uncommon. Thorough evaluation of maternal etiology is important given the potential risk of undiagnosed chronic gastritis and even more of the autoimmune gastritis (formerly called pernicious anemia), a premalignant condition for gastric cancer. In our cohort, we indirectly identified, via the newborns, three mothers with previously unrecognized chronic gastritis; both had metaplastic changes, and one was subsequently diagnosed with early malignancy. Our data suggest that newborn screening may also enable earlier diagnosis of chronic gastritis and other causes of B12 malabsorption in otherwise asymptomatic mothers.

Our study has two limitations. Firstly, milder forms of B12 deficiency may not be detectable at birth and may reduce primary care pediatricians’ awareness of B12 deficiency. Secondly, because our pilot was conducted only in Prague—an area with a relatively higher socioeconomic profile and excellent prenatal care—the nationwide birth frequency may be even higher in regions outside Prague and other major cities where these conditions are less favorable.

Implementation of B12 deficiency screening into the routine Czech NBS program appears clinically well justified, provided that an appropriate approval will be granted by governing bodies and payers. Scaling up B12 screening may require a higher second-tier testing capacity and a broader recall network (e.g., involving regional centers) to ensure timely and equitable follow-up.

## 5. Conclusions

In this Czech pilot NBS program for neonatal B12 deficiency, 34,384 newborns were screened using routine DBS sampling with a first-tier C3-based algorithm and second-tier MMA testing on the same DBS. Eight newborns met the case definition, corresponding to a birth frequency of 1:4228 (95% CI 1:2176–1:9931), which is comparable to published European data and similar to HPA/PKU birth frequency in the Czech Republic. The strategy was economically and operationally feasible within the existing screening workflow (no additional blood draw; second-tier testing performed on the original DBS) and improved diagnostic precision: the PPV for confirmed disease increased from 0.59% for first-tier results to 88.89% for a positive second-tier MMA result, with a low FPR of 0.0029%.

From a public health perspective, implementation of NBS for B12 deficiency appears inexpensive and clinically well justified owing to simple and effective treatment. In addition, neonatal case-finding has a secondary benefit for the mothers by identifying previously unrecognized B12 deficiency. Targeted maternal management can improve the infant’s ongoing B12 supply during breastfeeding and reduce maternal morbidity by identifying clinically silent chronic gastritis. Neonatal B12 deficiency screening may have benefits beyond the health of newborns.

## Figures and Tables

**Table 1:** Results of primary markers and second-tier tests in original DBS of recalled newborns. The upper or lower reference limit is shown in parentheses for each parameter (as applicable). C3—propionylcarnitine; C3/C2—propionylcarnitine/acetylcarnitine ratio; C3/Met—propionylcarnitine/methionine ratio; FP—false positive; MMA—methylmalonic acid; TP—true positive; tHcy—total homocysteine; **bold**—pathological values.

No.	C3 µmol/L (<3.8)	C3/C2 (<0.3)	Met µmol/L (>7)	C3/Met (<0.50)	MMA µmol/L (<2.5)	tHcy µmol/L (<12)	Subsequent Confirmatory Testing
1	**5.63**	0.21	17.20	0.32	**24.60**	**40.70**	**TP**
2	**3.80**	0.17	17.30	0.22	**3.50**	6.70	FP
3	**7.10**	0.12	26.00	0.27	**5.00**	**20.50**	**TP**
4	**3.97**	0.13	17.60	0.23	**5.40**	**26.80**	**TP**
5	**4.70**	0.11	27.60	0.23	**4.60**	**15.00**	**TP**
6	**4.30**	0.17	23.00	0.19	**6.20**	8.20	**TP**
7	**4.90**	0.13	19.00	0.26	**4.00**	10.30	**TP**
8	**5.20**	0.13	16.00	0.32	**4.50**	**19.80**	**TP**
9	**3.90**	0.12	21.00	0.19	**6.30**	11.10	**TP**

**Table 2:** Confirmatory testing results of newborns and mothers. Abnormally low values are indicated in blue, whereas values above the cut-off are indicated in red. AA—adequate absorption; BA—borderline absorption; cB12—combined B12; CS—CobaSorb; D—day; d—days; def.—deficient; Ery—erythrocytic; fol—folates; FP—false positive; HoloTC—active B12; M—mother; MA—malabsorption; MMA—methylmalonic acid; N—newborn; N/A—not available; NC—non-compliance; S—serum; tHcy—total homocysteine; TP—true positive; y—years; light orange—CobaSorb results.

										CobaSorb						
No.	N M	Age	Holo TC	Total B12	MMA	tHcy	cB12	S-fol	Ery-fol	D1	D3	D3-D1	D3/D1	CS	M B12 def.	Confirmation
Cut-Offs		d/y	pmol/L		nmol/L	µmol/L		µg/L		pmol/L			Ratio			
			<31	<148	N > 700 M > 271	N > 10 M > 15	<−1.5	<3.9	<523			<10	<1.22			
1	N	14	2	51	32,900	126.3	−5.4			2	40	47	24.5	AA		TP
	M	40.7	5	84	3250	42.5	−3.3	36.0	1067.6	5	8	3	1.6	MA	**Yes**	
2	N	14	53	137	472	8.2	−0.5	19.8		53	124	71	2.3	AA		FP
	M	25.3	68	338	221	12.2	0.2	13.5	1133.4	68	170	102	2.5	AA	No	
3	N	25	5	74	6820	32.2	−3.6	12.6		5	80	75	16.0	AA		TP
	M	29.4	24	NA	1930	15.5	NA	NA	NA	24	N/A	N/A	N/A	N/A	Yes	
4	N	13	3	74	1550	35.9	−3.2	16.0		3	62	59	20.7	AA		TP
	M	37.0	10	134	1230	19.6	−2.0	13.0	1004.4	10	22	12	2.2	BA	Yes	
5	N	23	>256	675	2830	11.7	NA	NA	907.9	>256	N/A	N/A	N/A	NC		TP
	M	28.9	>256	545	231	8.8	1.1	4.1	1014.1	>256	N/A	N/A	N/A	NC	No	
6	N	10	30	74	9430	17.4	−2.7	NA		30	202	172	6.7	AA		TP
	M	24.8	48	219	270	8.5	−0.1	5.6	1432.8	48	256	208	5.3	AA	No	
7	N	21	21	107	1940	17.9	−2.0	11.9		21	87	66	4.1	AA		TP
	M	35.1	59	189	199	20.6	−0.3	3.5	682.3	59	99	40	1.7	AA	No	
8	N	15	4	74	17,500	43.5	−4.2	19.9		4	42	38	10.5	AA		TP
	M	36.9	10	138	2590	15.1	−2.2	14.5	955.8	10	20	10	2.0	MA	Yes	
9	N	20	18	100	1910	19.9	−2.1	13.3		18	109	91	6.1	AA		TP
	M	38.9	48	201	430	11.9	−0.5	2.8	899.8	48	115	67	2.4	AA	No	

**Table 3:** Confirmatory testing results of newborns and mothers. Abnormally low values are indicated in blue, whereas values above the cut-off are indicated in red. FFQ—Food Frequency Questionnaire; GPCAb—gastric parietal cell antibodies; IF—intrinsic factor; M—mother; N/A—not available; Pep—pepsinogen.

No.	FFQ M	B12 Supp. During Pregnancy	Fol Supp. During Pregnancy	GPCAb	Anti IF	Gastrin	Pep 1	Pep1/ Pep2	Anti H^+^/K^+^	Etiology	Biopsy in M If Performed
Ref.		µg/kg/d	µg/kg/d		U/mL	pmol/L	µg/L	%	U/mL		
Range				Neg.	0–6.0	1.7–7.6	28.0–158.0		0–7.0		
1	Unknown	Unknown	Unknown	pos.	0.7	269.7	7.9	0.9	N/A	Chronic gastritis	Referred for gastroscopy, but did not attend
2	Omnivore	Unknown	Unknown	neg.	0.6	6.5	36.9	6.0	N/A	False positive	
3	Vegetarian	0.0	0.0	N/A	N/A	N/A	N/A	N/A	N/A	Lifelong vegan	
4	Omnivore	0.0	0.0	pos.	1.5	291.3	49.0	8.8	N/A	Chronic gastritis	Autoimmune gastritis, later diagnosed with a malignancy
5	Omnivore	Unknown	Unknown	neg.	0.9	3.2	68.0	3.7	N/A	Unknown	
6	Omnivore	0.1	11.8	neg.	<0.5	33.0	62.8	3.7	N/A	Unknown	
7	Omnivore	0.1	11.3	neg.	<0.5	2.9	37.1	11.6	N/A	Unknown	
8	Omnivore	0.1	12.7	pos.	0.7	132.1	7.5	0.7	107.0	Chronic gastritis	Autoimmune metaplastic atrophic gastritis
9	Omnivore	0.0	0.0	neg.	<0.5	3.1	44.3	6.2	0.4	Unknown	

## Data Availability

The original contributions presented in this study are included in the article/Supplementary Materials. Further inquiries can be directed to the corresponding author.

## Supplementary Materials

The following supporting information can be downloaded at: https://www.mdpi.com/article/10.3390/ijns12020030/s1, Supplementary Material S1: FFQ, Supplementary Material S2: B12 prescription.

## Abbreviations

The following abbreviations are used in this manuscript:

AI	adequate intake
anti-IF	anti-intrinsic factor
B12	vitamin B12
CI	confidence interval
CV	coefficient of variation
C3	propionylcarnitine
C3/C2	propionylcarnitine/acetylcarnitine ratio
C3/Met	propionylcarnitine/methionine ratio
Cbl	cobalamin
DBS	dried blood spot
FFQ	Food Frequency Questionnaire
FIA–MS/MS	flow-injection tandem mass spectrometry
FPR	false positive rate
GPCAb	gastric parietal cell antibodies
HPA/PKU	Hyperphenylalaninemia/phenylketonuria
holoTC	holotranscobalamin
LC–MS/MS	liquid chromatography–tandem mass spectrometry
Met	methionine
Met/Phe	methionine/phenylalanine ratio
MMA	methylmalonic acid
MRM	multiple-reaction monitoring
MTHFR	methylenetetrahydrofolate reductase
NBS	newborn screening
PKU	phenylketonuria
PPV	positive predictive value
tHcy	total homocysteine
